# Smoking and liver diseases: an updated review of pathogenesis, progression, and therapeutic implications

**DOI:** 10.1007/s10238-025-01922-6

**Published:** 2025-12-03

**Authors:** Gasser El-Azab, Ehab Elkhouly, Rania Abouyoussef, Hanaa Nagdy

**Affiliations:** 1 Hepatology and Gastroenterology Department, National Liver Institute, Menoufia University, Shebeen El-Kom, Egypt; 2 Tropical Medicine Department, Faculty of Medicine, Alexandria University, Alexandria, Egypt; 3 Internal Medicine Department, College of Medicine, Arab Academy for Science and Technology and Maritime Transport, Alexandria, Egypt

**Keywords:** Smoking, Liver diseases, Hepatocellular carcinoma, Fibrosis, MASLD, E-cigarettes, Smoking cessation

## Abstract

Cigarette smoking, a leading cause of preventable morbidity and mortality worldwide, has increasingly been recognized as a significant and independent risk factor for the development and progression of various liver diseases. Historically, the direct impact of smoking on liver health received limited attention compared to its well-established effects on the respiratory and cardiovascular systems. However, a growing body of evidence now unequivocally demonstrates that smoking negatively influences the incidence, severity, and outcomes of a wide spectrum of hepatic conditions, including metabolic dysfunction-associated steatotic liver disease (MASLD), alcohol-related liver disease (ALD), chronic viral hepatitis (HBV and HCV), primary biliary cholangitis (PBC), and hepatocellular carcinoma (HCC). The detrimental effects of tobacco extend to patients undergoing liver transplantation, where smoking is associated with increased post-transplant complications and mortality. The underlying mechanisms are complex, involving direct and indirect toxic effects, immunologic dysregulation, and oncogenic pathways, primarily driven by oxidative stress, systemic inflammation, insulin resistance, and the presence of numerous carcinogens in tobacco smoke. This comprehensive review synthesizes current knowledge, highlighting the multifaceted ways in which smoking impacts liver health, from cellular injury and fibrosis progression to increased cancer risk and compromised transplant outcomes. In addition, we explore the rising prevalence of electronic cigarette use and present the latest evidence regarding their potential impact on liver health. We emphasize the critical importance of smoking cessation as a therapeutic intervention across all stages of liver disease and discuss the challenges and strategies for its implementation. By integrating the updated research data and clinical insights, this review aims to underscore the urgent need for greater awareness among healthcare professionals and patients regarding the profound and pervasive link between smoking and liver disease, advocating for targeted interventions to alleviate this preventable burden.

## Introduction

Tobacco smoking stands as a formidable global health challenge, recognized as a leading cause of preventable morbidity and mortality. The World Health Organization estimates that approximately one-third of the world's male population smokes tobacco, and cigarette smoking was responsible for an estimated 100 million deaths worldwide in the twentieth century [1]. While the profound adverse effects of smoking on the respiratory and cardiovascular systems are extensively documented and widely acknowledged, its direct and indirect impact on liver health has historically received comparatively less attention from the medical community, particularly hepatologists. This oversight may have contributed to a misconception that smoking, in itself, is not a primary causative agent in chronic liver disease [2].

However, a paradigm shift has occurred in recent years, with mounting evidence unequivocally demonstrating the deleterious effects of cigarette smoking across the spectrum of liver diseases. Tobacco substances undergo first-pass metabolism in the liver, exposing the organ to a complex array of over 4000 toxic chemicals, including nicotine, nitrosamines, aromatic hydrocarbons, and various carcinogens [2–4]. These compounds initiate and perpetuate a cascade of pathological processes within the liver, contributing to the incidence, progression, and severity of numerous hepatic conditions.

This comprehensive review aims to synthesize the current understanding of the intricate relationship between smoking and liver diseases. We will delve into the multifaceted mechanisms by which tobacco exposure inflicts hepatic injury, ranging from direct cellular toxicity and immunologic dysregulation to oncogenic transformation. Furthermore, we will systematically examine the specific impact of smoking on key liver pathologies, including metabolic dysfunction-associated steatotic liver disease (MASLD), alcohol-related liver disease (ALD), chronic viral hepatitis, primary biliary cholangitis (PBC), and hepatocellular carcinoma (HCC). The review will also address the critical implications of smoking in the context of liver transplantation, highlighting its influence on post-transplant outcomes. Throughout this discussion, we will incorporate updated research data and clinical insights to provide a contemporary perspective on this evolving field. Ultimately, this review seeks to underscore the urgent necessity for heightened awareness and proactive interventions aimed at promoting smoking cessation as a pivotal strategy in the prevention and management of liver diseases globally.

## Mechanisms of liver injury induced by smoking

The detrimental effects of cigarette smoking on the liver are mediated through a complex interplay of toxic, immunologic, and oncogenic mechanisms. The vast array of chemical constituents in tobacco smoke, including nicotine, nitrosamines, aromatic hydrocarbons, vinyl chloride, and cadmium, plays a central role in these pathological processes [4].

### Toxic effects

Smoking exerts both direct and indirect toxic effects on hepatic cells. Directly, many substances in cigarette smoke possess cytotoxic properties that induce oxidative stress, a critical pathway in liver injury and fibrosis [5]. This involves the increased production of reactive oxygen species (ROS) and a concomitant impairment of antioxidant defenses, such as glutathione [6, 7]. The resulting lipid peroxidation further damages hepatocytes, leading to inflammation and cell death. This cellular damage activates hepatic stellate cells (HSCs), which are key players in liver fibrogenesis, contributing to the deposition of extracellular matrix proteins and the progression of fibrosis [8–10].

Indirectly, smoking can lead to secondary polycythemia due to increased carboxyhemoglobin levels, which reduces the oxygen-carrying capacity of the blood and results in tissue hypoxia [11, 12]. This hypoxia, coupled with increased erythropoietin production, can lead to a rise in red cell mass and subsequent iron accumulation in hepatocytes [13]. Iron deposition is a known mechanism of liver injury, promoting oxidative stress and contributing to fibrosis, particularly observed in heavy smokers with chronic hepatitis C [14–17]. Furthermore, smoking induces vascular vasoconstriction, sinusoidal endothelial dysfunction, and smooth muscle proliferation, leading to hypoxia, capillarization of sinusoids, and further activation of HSCs [18].

### Immunologic effects

Smoking profoundly impacts both innate and adaptive immunity, often aggravating pathological immune responses while suppressing defensive mechanisms [19]. Nicotine, a major component of tobacco, has been shown to inhibit lymphocyte proliferation and differentiation, leading to suppressed antibody formation [20]. Smoking also induces apoptosis of lymphocytes, increases CD8+ cytotoxic T cells, decreases CD4+ cells, and impairs natural killer cell activity [21, 22]. These changes can be reversed within a month of smoking cessation, highlighting the direct impact of tobacco on immune cell function [23].

**Fig. 1:**
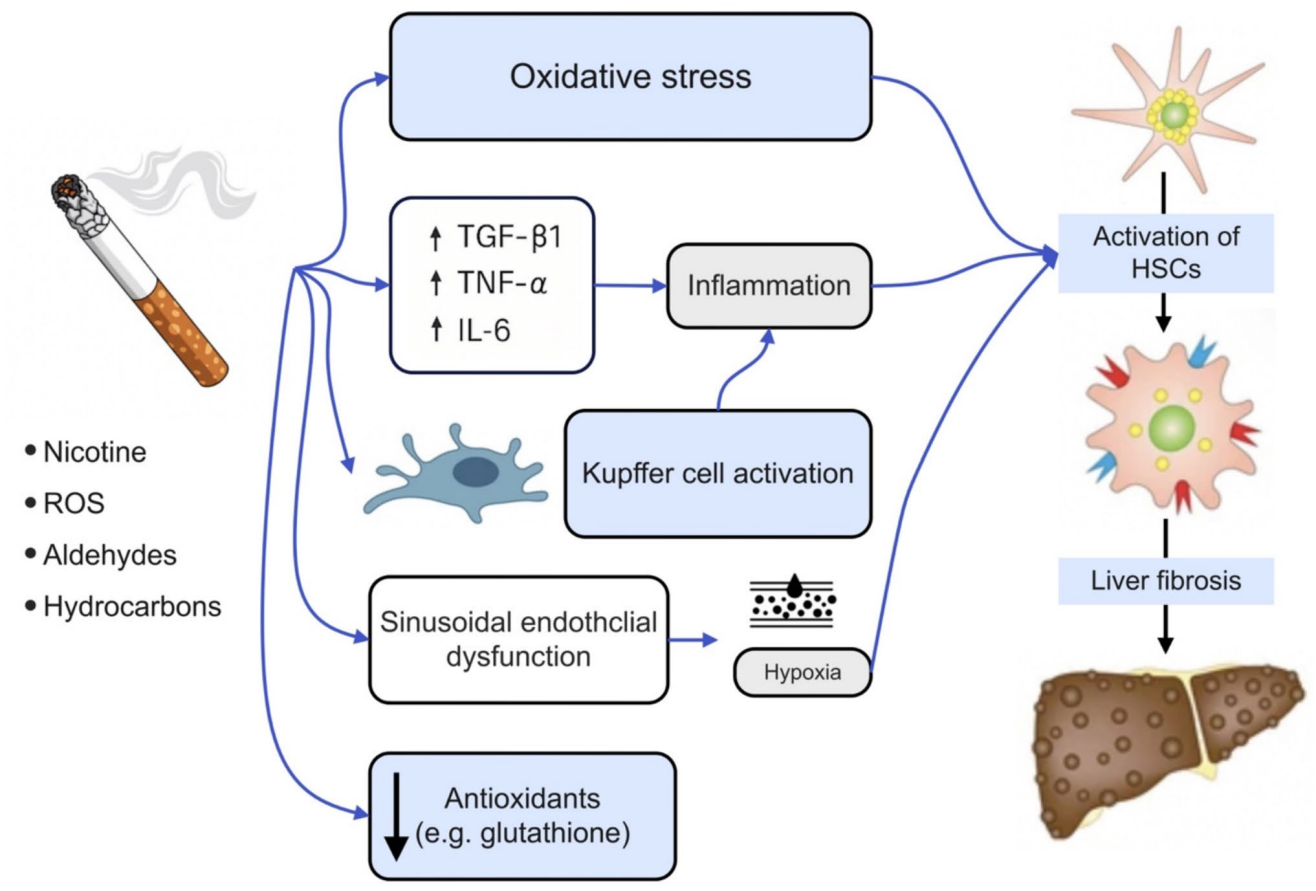
Mechanisms by which smoking promotes liver fibrosis. Cigarette smoking contributes to hepatic fibrosis through multiple interrelated pathways. Components of cigarette smoke, such as nicotine and reactive oxygen species (ROS), induce oxidative stress, stimulate the production of inflammatory cytokines (TNF-α, IL-6, and TGF-β1), and directly activate hepatic stellate cells (HSCs). Additionally, smoking promotes sinusoidal endothelial dysfunction, depletes antioxidant defenses, and alters immune cell function, all of which converge to increase extracellular matrix (ECM) deposition and fibrosis progression

At a molecular level, smoking affects key signaling pathways such as nuclear factor-kappa B (NF-kB) and mitogen-activated protein kinases (MAPK) and induces epigenetic modifications like DNA methylation and histone modification [22]. These alterations contribute to systemic inflammation by increasing the production of pro-inflammatory cytokines, including interleukin (IL)-1, IL-6, IL-8, IL-13, tumor necrosis factor-alpha (TNF-α), and transforming growth factor-beta 1 (TGF-β1) [24]. This chronic inflammatory state plays a central role in liver injury and the progression of liver disease. Among these mediators, TGF-β1 is particularly significant, as it directly activates HSCs and stimulates the synthesis of extracellular matrix proteins, thereby promoting fibrogenesis. Figure 1 illustrates the mechanisms by which smoking promotes liver fibrosis.

The dysregulation of the immune system by smoking can also contribute to autoimmune diseases, and it creates conflicting regulatory effects on T regulatory cells, potentially predisposing individuals to infections [25].

### Oncogenic effects

The oncogenic potential of cigarette smoke is well-established and operates through a multifaceted process. Several carcinogens in tobacco smoke are directly implicated in liver cancer development. Among these, N-nitrosodimethylamine (NDMA) is a potent hepatocarcinogen that induces DNA alkylation, oxidative stress, and promotes liver fibrosis, ultimately facilitating HCC development [26]. Similarly, 4-aminobiphenyl, metabolized by hepatic CYP1A2, produces reactive intermediates that form DNA adducts in hepatocytes, increasing the mutational burden [27]. Other constituents, such as vinyl chloride, have a well-established association with hepatic angiosarcoma [28], whereas cadmium enhances liver cancer risk through proto-oncogene activation, oxidative stress induction, and stimulation of pro-inflammatory cytokines [29–31].

The molecular pathways linking smoking to HCC are complex and involve genotoxicity, epigenetic alterations, and disruption of tumor-suppressor functions. Tobacco carcinogens cause direct DNA damage, including adduct formation, and amplify the mutagenic effect of other hepatotoxins such as aflatoxin B1 [32, 33]. Additionally, tobacco exposure downregulates p53, a critical tumor suppressor gene, facilitating genomic instability and malignant transformation [34].

Recent evidence also demonstrates the dual role of nicotine, which primarily promotes hepatocarcinogenesis through activation of multiple oncogenic signaling cascades, including α7-nicotinic acetylcholine receptor (α7-nAChR)/JAK2/STAT3, CYP1A1/PI3K/Akt signaling mediated by NF-κB and AP-1, SETDB1/CDK6-mediated STAT1 stabilization, GCF2-driven Wnt/β-catenin/SOX2 signaling, and the β2-adrenergic receptor (β2-AR)/PI3K/Akt pathway [35–37]. Conversely, nicotine may exert paradoxical inhibitory effects by activating immune pathways and inducing apoptosis, such as through stimulation of immature dendritic cells via PI3K/Akt signaling and modulation of caspase-3 and Cyclin B1 expression through α7-nAChR signaling [36]. Comprehensive genomic analyses have revealed a distinct tobacco-related mutational signature in liver tumors of smokers, characterized by cytosine-to-adenine base substitutions, dinucleotide alterations, and guanine adduct formation, patterns resembling those observed in lung and head-and-neck cancers [38–40]. Moreover, telomerase reverse transcriptase (TERT) promoter mutations, strongly associated with smoking, have been implicated in hepatocarcinogenesis and tumor progression [41–43].

Host genetic susceptibility further modifies the oncogenic impact of smoking. For instance, polymorphisms in inflammatory genes, such as interleukin-1β -31T/C, interact synergistically with tobacco exposure and alcohol intake to elevate HCC risk [44].

### Systemic effects

Beyond direct hepatic impact, smoking exerts systemic effects that indirectly contribute to liver disease progression. Chronic exposure to cigarette smoke leads to persistent systemic inflammation, which is a key driver of liver injury [45]. Furthermore, smoking is strongly associated with insulin resistance and visceral fat accumulation, both of which are central to the pathogenesis of metabolic syndrome and MASLD [46]. The synergistic interaction between smoking and other metabolic risk factors, such as hypertension and dyslipidemia, further exacerbates cardiovascular disease risk, a major cause of mortality in patients with MASLD. The impaired wound healing response observed in smokers can also hinder hepatic regeneration, thereby exacerbating fibrosis and increasing the likelihood of replication errors and de novo mutations, potentially linking to carcinogenesis [47, 48].

## Smoking and specific liver diseases

### Metabolic dysfunction-associated steatotic liver disease

Among chronic liver disorders, MASLD is the most prevalent worldwide, with an estimated global prevalence of 20–39%, depending on region, diagnostic method, and population studied [49–51]. A growing body of evidence now strongly indicates that cigarette smoking significantly contributes to both the incidence and severity of MASLD [52–54].

The association between smoking and MASLD is underpinned by shared pathological mechanisms, particularly insulin resistance and visceral fat accumulation, which are central to the metabolic syndrome (MetS). While early cross-sectional studies yielded inconsistent results regarding this association [55–57], more robust prospective longitudinal cohort studies have provided compelling evidence. These studies, which adjust for time-changing confounders such as diet, physical activity, and alcohol intake, demonstrate a clear link between active smoking and incident MASLD [58, 59]. Importantly, a clear dose–response relationship has been documented, with the risk of MASLD increasing proportionally to cumulative smoking exposure. In men, studies have reported multivariable-adjusted hazard ratios (aHR) of 1.25 (95% CI 1.21–1.29) for those with 10–19.9 pack-years and 1.36 (95% CI 1.30–1.42) for those exceeding 20 pack-years compared with non-smokers. Among women, the corresponding aHRs (95% CI) for NAFLD were 1.25 (1.04–1.50) for 5–9.9 pack-years and 1.46 (1.17–1.81) for ≥ 10 pack-years, relative to non-smokers [60]. Recent evidence further reinforces this association, showing that cigarette smoking is significantly associated with increased odds of MASLD, with smoking cessation for more than 10 years demonstrating beneficial effects [52, 61].

Histological analyses of liver tissue provide some of the most convincing evidence for the link between smoking and MASLD progression. Advanced fibrosis at diagnosis has been independently associated with a smoking history of more than 10 pack, with an odds ratio (OR) of 1.63 (95% CI 1.19–2.24) [62]. Furthermore, studies evaluating the histological progression of MASLD severity over time using paired liver biopsies have found a lower likelihood of fibrosis regression among active smokers (e.g., only 4% regression compared to 12% progression) [63]. While the pro-fibrogenic effects of smoking are well-documented, it is worth noting that the impact of tobacco on the degree of steatohepatitis is less marked, with no significant differences in MASH resolution [64].

**Fig. 2:**
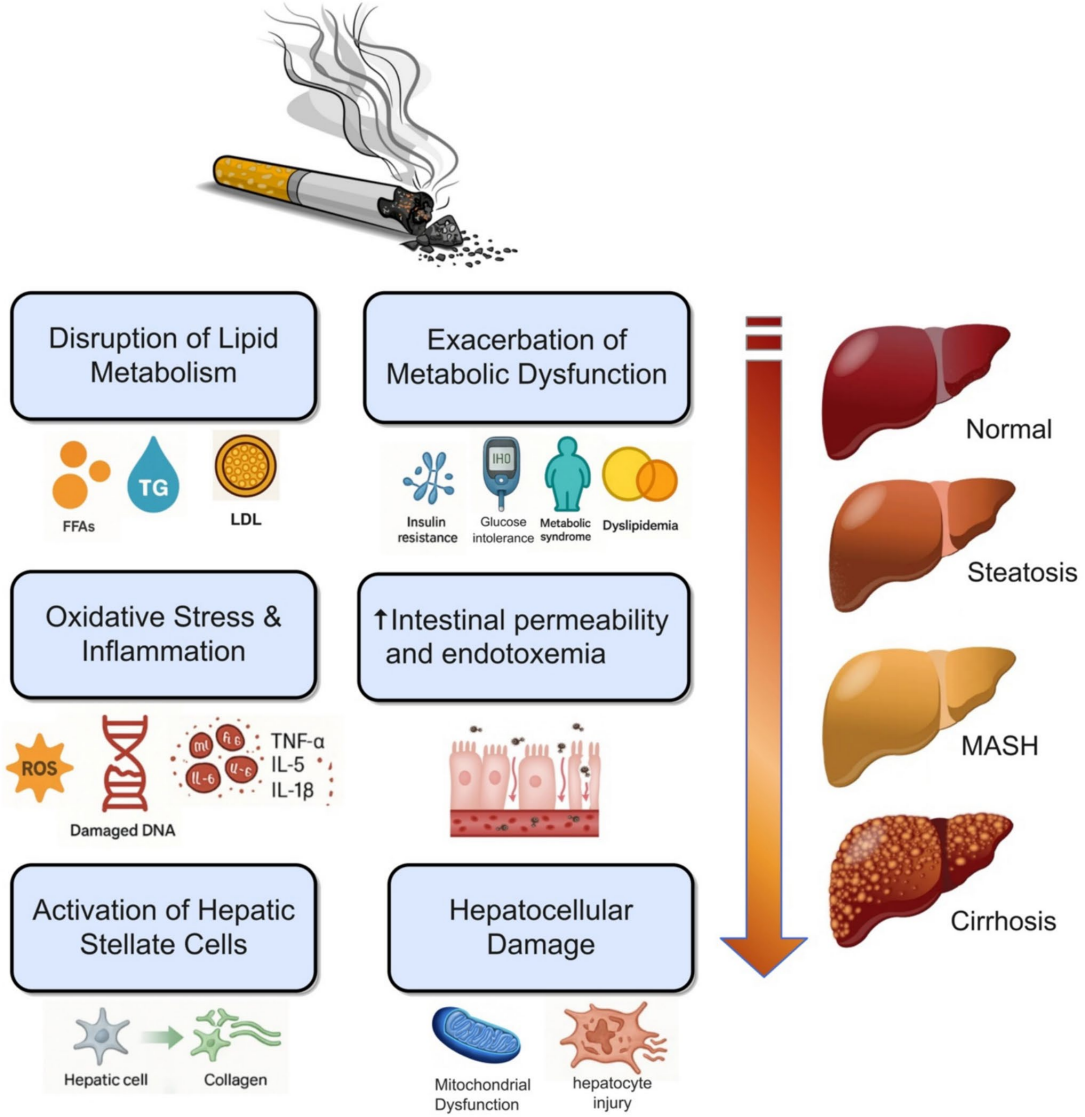
Mechanisms by which smoking promotes the development and progression of MASLD. This diagram illustrates the multifactorial mechanisms through which cigarette smoking contributes to MASLD. Smoking disrupts lipid homeostasis, leading to excessive hepatic triglyceride accumulation and increased LDL cholesterol and free fatty acids. It induces hepatocellular injury by promoting insulin resistance, mitochondrial dysfunction, ER stress, and apoptosis. Cigarette smoke generates reactive oxygen species (ROS), leading to oxidative damage to lipids, proteins, and DNA, while activating inflammatory pathways that increase cytokines such as TNF-α, IL-6, and IL-1β. These inflammatory signals, combined with ROS, stimulate hepatic stellate cells to produce extracellular matrix, driving fibrosis and cirrhosis progression. Smoking also exacerbates systemic metabolic dysfunction, worsening insulin resistance, dyslipidemia, and glucose intolerance. Additionally, it disrupts the gut–liver axis, increasing intestinal permeability and endotoxemia, which activates Kupffer cells and perpetuates liver inflammation. Collectively, these interrelated processes promote the transition from simple steatosis to steatohepatitis, advanced fibrosis, and cirrhosis in smokers with MASLD

Smoking promotes MASLD through several interrelated mechanisms. It induces insulin resistance, increasing hepatic fat accumulation via enhanced free fatty acid influx and de novo lipogenesis [65]. Smoking also triggers oxidative stress, mitochondrial injury, and chronic liver inflammation, which are central to disease progression [61, 66]. Additionally, it disrupts the gut–liver axis, leading to endotoxemia and Kupffer cell activation [67]. These effects, combined with smoking-induced epigenetic modifications and fibrogenesis [61], promote the transition from simple steatosis to MASH and liver fibrosis (Fig. 2).

Smoking not only contributes to the development of MASLD but also plays a significant role in the progression of liver fibrosis. Multiple cross-sectional and longitudinal studies have confirmed this association. A large biopsy-based study reported that a smoking history of ≥ 10 pack-years was significantly associated with advanced fibrosis (adjusted OR 1.63; 95% CI 1.19–2.24) [62]. Similarly, a Korean cohort of 199,468 adults demonstrated a positive, dose–response relationship between smoking exposure and both incident MASLD and fibrosis severity. Men with ≥ 20 pack-years had an aHR of 1.29 (95% CI 1.18–1.42), while women with ≥ 10 pack-years had an aHR of 1.75 (95% CI 1.12–2.73) for MASLD with significant fibrosis [60].

Other smaller studies support these findings. Ou et al. observed that smokers with MASLD had significantly higher liver stiffness values than nonsmokers (10.12 ± 10.38 vs. 7.26 ± 6.42 kPa, *P* = 0.013), and smoking remained an independent predictor of fibrosis (OR = 1.294, *P* = 0.015) even after adjusting for confounders [62]. Furthermore, fibrosis severity correlated with smoking index and coexistent diabetes, reinforcing a dose-dependent effect. Overall, these results indicate that greater cumulative smoking exposure significantly increases both the risk and severity of liver fibrosis in MASLD. Nevertheless, most studies relied on ultrasonography or noninvasive fibrosis markers, which may underestimate true prevalence and histological severity.

Cardiovascular disease (CVD) remains the leading cause of mortality among patients with MASLD [68–70]. Smoking significantly exacerbates this risk, with recent evidence showing that cigarette smoking is associated with a 22% increased risk of CVD events in MASLD patients compared to non-smokers (aHR 1.22, 95% CI 1.15–1.30) [71]. Unlike its additive effect with diabetes on MASH-fibrosis, smoking exhibits a synergistic effect with other metabolic risk factors, such as hypertension and dyslipidemia, on the development of CVD [72–74]. This biological link is supported by common underlying pathways, such as hepatic and systemic insulin resistance, atherogenic lipid profiles, hypertension, and increased expression of pro-atherogenic, pro-coagulant, and pro-inflammatory factors [75].

### Alcohol-related liver disease

The relationship between alcohol consumption and liver disease is well-established. Yet, the interplay between smoking and alcohol in the context of liver injury is increasingly recognized as a critical area of study. Patients with alcohol misuse are significantly more likely to smoke heavily, with some estimates suggesting a threefold higher prevalence compared to the general population. Conversely, the prevalence of alcohol abuse is approximately fourfold higher among individuals with nicotine dependence [76]. This strong co-occurrence underscores the importance of understanding their combined impact on liver health.

While the precise extent to which cigarette smoking aggravates the clinical course of ALD remains an area of ongoing research, emerging evidence suggests a synergistic effect. Smoking may exacerbate the pathogenic effects of alcohol on the liver, implying that the combined exposure to both substances leads to more severe liver damage than either factor alone [77]. Clinical data indicate that smoking adversely affects multiple aspects of liver disease, including the incidence and severity of hepatic steatosis, progression of fibrosis, development of HCC, and outcomes among alcohol-related liver transplant recipients [78, 79]. A recent study further demonstrated that smoking increased the risk of ALD, cirrhosis, and HCC by 1.32-, 1.53-, and 1.53-fold, respectively (all *p* < 0.001). Gender-specific analysis revealed significantly higher risk ratios for women, particularly among those who smoked and engaged in high-risk drinking behavior [80]. Additionally, a large Danish population-based study reported that women who smoked more than 10 g of tobacco per day had an aHR of 3.9 (95% CI 1.6–9.4) for developing alcoholic cirrhosis, whereas men had an aHR of 2.2 (95% CI 1.4–3.4). For reference, one cigarette was considered equivalent to 1 g of tobacco, one cheroot or pipe to 3 g, and one cigar to 5 g. Similar associations were observed for all-cause cirrhosis [81]. In a cohort of 128,934 individuals, Klatsky and Armstrong found that smoking one or more packs of cigarettes per day was associated with a threefold increase in the risk of developing alcoholic cirrhosis compared to non-smokers [82].

The mechanisms underlying this synergy are complex. Both alcohol and smoking contribute to systemic inflammation, oxidative stress, and insulin resistance, creating a highly detrimental environment for the liver [77]. The presence of numerous toxins and carcinogens in tobacco smoke, combined with the metabolic burden of alcohol, can overwhelm the liver's detoxification pathways and promote progressive injury. This includes enhanced fibrogenesis and an increased risk of developing alcoholic hepatitis and ultimately cirrhosis [4]. Earlier data also highlight that smoking status impacts various clinical outcomes in metabolic dysfunction and alcohol-associated liver disease and that smoking, along with obesity and metabolic syndrome, contributes significantly to ALD risk [83]. These findings emphasize the need for integrated approaches to address both alcohol and tobacco use in patients with ALD to improve clinical outcomes.

### Viral hepatitis

The impact of cigarette smoking on the progression and outcomes of chronic viral hepatitis, specifically Hepatitis B virus (HBV) and Hepatitis C virus (HCV) infections, is a critical area of research. While the data may sometimes appear scarce, existing studies consistently point to a detrimental role of smoking in these conditions [2].

Smoking has been associated with increased disease progression in patients with chronic viral hepatitis. For instance, studies have shown that cigarette smoking, particularly heavy smoking (e.g., > 1 pack/day), is associated with elevated alanine aminotransferase (ALT) levels in HCV antibody-positive patients [16]. This suggests that smoking contributes to ongoing liver inflammation in these individuals. Furthermore, a synergistic effect between smoking and alcohol consumption on elevated ALT levels has been observed in HCV patients, indicating that the combined exposure significantly exacerbates liver injury [84]. While some studies have not found a similar effect in Hepatitis B surface antigen (HBsAg)-seropositive patients regarding ALT levels, other research indicates an increased risk of cirrhosis in HBV carriers who smoke 20 or more cigarettes per day, an effect that is particularly pronounced when combined with habitual alcohol consumption [85, 86].

Beyond inflammation and fibrosis progression, smoking has also been linked to lower response rates to antiviral therapy in HCV infection [87, 88]. This highlights a significant clinical implication, as smoking could undermine the effectiveness of crucial treatments aimed at eradicating the virus and preventing disease progression. Studies have demonstrated that smoking is associated with increased fibrosis and inflammation activity scores on liver biopsy in HCV patients [16, 89, 90]. The pro-inflammatory effects of smoking likely contribute to this accelerated progression of liver damage in the context of viral hepatitis.

It is important to note that the complex interplay between smoking, viral hepatitis, and other cofactors like alcohol necessitates the further research. However, the available evidence strongly suggests that smoking acts as an independent risk factor that exacerbates the severity and progression of chronic viral hepatitis, increasing the risk of advanced fibrosis and cirrhosis, and potentially impacting treatment efficacy. Therefore, smoking cessation should be a key component of management strategies for patients with chronic HBV and HCV infections.

### Primary biliary cholangitis

A growing body of evidence suggests that cigarette smoking is associated with an increased risk of developing PBC. A comprehensive meta-analysis of nine case–control studies involving over 21,000 participants demonstrated that individuals who had ever smoked had a significantly higher risk of PBC compared to never-smokers (pooled OR: 1.31; 95% CI 1.03–1.67). Subgroup analysis revealed that former smokers exhibited the highest risk (pooled OR: 1.36; 95% CI 1.01–1.84), whereas current smokers showed a nonsignificant increase (pooled OR: 1.18; 95% CI 0.94–1.50) [91]. Earlier analyses supported this association, reporting a stronger relationship between smoking and PBC (pooled OR: 1.67; 95% CI 1.41–1.92) across different populations [92]. Notably, a Korean case–control study identified both active and passive smoking as independent risk factors for PBC (OR: 2.03; 95% CI 1.06–3.93) [93], while a large multicenter Japanese study also confirmed that ever-smoking significantly increased the risk of PBC (OR: 1.70; 95% CI 1.28–2.25) [94].

Beyond disease onset, smoking appears to negatively influence disease course in PBC. A pooled analysis of three cross-sectional studies (*n* = 544) reported that ever-smokers had a threefold higher risk of advanced liver fibrosis compared to non-smokers (pooled OR: 3.00; 95% CI 1.18–7.65) [95]. Another study demonstrated that active and passive smoking were linked to more advanced histological stage and higher mortality in PBC patients; importantly, cumulative exposure (measured in pack-years) was strongly associated with fibrosis severity, with each additional pack-year tripling the likelihood of advanced fibrosis (adjusted OR per pack-year: 3.2; 95% CI 2.02–6.29) [10]. Smoking has also emerged as a major risk factor for hepatocellular carcinoma (HCC) in PBC, with current smokers showing more than a twofold increased risk of HCC (adjusted HR: 2.31; 95% CI 1.47–3.63) [96].

Collectively, these findings underscore that cigarette smoking is not only associated with an increased risk of developing PBC but also accelerates disease progression, worsens fibrosis, elevates HCC risk, and contributes to poorer long-term outcomes.

### Hepatocellular carcinoma

HCC stands as the most common primary liver cancer and is a leading cause of cancer-related deaths worldwide, with its incidence continuing to rise [97]. While chronic infections with HBV and HCV, heavy alcohol consumption, and diabetes are well-established primary risk factors for HCC, there is compelling and growing evidence that prolonged and active cigarette smoking is an independent and significant risk factor for its development [98, 99]. The International Agency for Research on Cancer has officially recognized smoking as a risk factor for HCC [100].

Numerous epidemiological studies, including large meta-analyses of both cohort and case–control studies, have consistently demonstrated a strong association between cigarette smoking and an increased incidence of HCC. One meta-analysis, after adjusting for HBV infection, HCV infection, and alcohol consumption, found that current cigarette smoking increased the risk of HCC with a relative risk (RR) of 1.51 [101]. Another meta-analysis reported that smoking alone nearly doubled the risk of HCC [102]. In patients with HBV infection, smoking further increased the HCC risk by 1.4-fold, and even more dramatically, in individuals with HCV infection, the risk of HCC was increased 2.9-fold if there was a history of cigarette smoking [102].

The combined impact of smoking with other major risk factors is particularly significant. Evidence indicates that alcohol consumption, tobacco use, and obesity act synergistically, amplifying the risk of developing HCC [103]. Furthermore, current smokers have been shown to have a higher HCC risk (HR = 2.46, 95% CI = 1.77–3.43) in a dose-dependent manner with the number of cigarettes smoked [104].

Smoking also impacts HCC-related mortality. Heavy smoking has been linked to increased early mortality risk in HCC patients [105]. Data from a systematic review of 81 epidemiological studies indicate that alcohol and smoking are independent risk factors for worse overall and HCC-specific mortality [106]. Given this compelling evidence, controlling for smoking exposure is a prudent approach to HCC prevention, especially in patients with chronic viral hepatitis infections.

## Smoking and liver transplantation

Liver transplantation (LT) represents a lifesaving intervention for patients with end-stage liver disease, acute liver failure, and hepatocellular carcinoma. However, the success of LT can be significantly influenced by various pre- and post-transplant factors, among which cigarette smoking has emerged as a critical determinant of patient and graft outcomes. Patients undergoing evaluation for LT are particularly vulnerable to the systemic effects of tobacco, including its cardiovascular and renal implications [107].

Smoking is associated with a heightened risk of complications following liver transplantation. Transplanted patients who smoke face an increased likelihood of developing vascular complications, such as hepatic artery thrombosis, which can severely compromise graft function and patient survival [108]. Furthermore, smoking significantly elevates the risk of de novo malignancies post-transplant, including extrahepatic cancers, which contribute to long-term morbidity and mortality [109–111]. This increased cancer risk is a major concern, given the immunosuppressive regimens required post-LT, which can further predispose patients to oncogenesis [112].

The impact of smoking extends to overall patient and graft survival. Active smokers have consistently demonstrated increased mortality rates post-liver transplantation, often due to non-graft-related causes [107]. Recent studies have reinforced this, showing that current smokers have a 2.17-fold increase in the risk of death compared to non-smokers in the context of LT [113]. The probability of graft failure is also increased in active tobacco smokers at the time of liver transplantation [108]. Cardiovascular disease remains a predominant cause of death among LT recipients, and smoking significantly contributes to this risk [107, 114]. A dose-dependent effect has also been observed, with a 7% increase in the overall risk of death for every five pack-years increase in smoking history [113].

Beyond the direct physiological effects, smoking can also serve as a predictor of alcohol relapse in patients undergoing LT for alcohol-related liver disease [115]. This highlights a crucial behavioral aspect that needs to be addressed comprehensively in the pre-transplant evaluation and post-transplant follow-up [116]. Given these profound negative impacts, smoking cessation is considered a paramount intervention for candidates and recipients of liver transplantation.

## Electronic cigarette exposure and liver disease

Electronic nicotine delivery systems (ENDS), commonly known as e-cigarettes or vaping devices, have gained widespread popularity, particularly among adolescents and young adults, due to the perception that they are safer than conventional cigarettes [117]. While much research has focused on the cardiovascular and pulmonary effects, their impact on the liver, a key organ for metabolism and detoxification, is an emerging area of concern [118].

### Overview of e-cigarettes and toxicity

Electronic cigarettes (e-cigarettes) are battery-operated devices that heat a liquid solution, commonly referred to as e-liquid or vape juice, to create an inhalable aerosol. These e-liquids primarily consist of propylene glycol (PG) and vegetable glycerin (VG) as base solvents, nicotine in varying concentrations, and numerous flavoring additives such as diacetyl and cinnamaldehyde [119].

Upon heating, these components undergo thermal degradation, producing harmful by-products. The primary solvents, PG and VG, act as carriers for nicotine and flavorings, but when exposed to high temperatures, they decompose into reactive carbonyl compounds such as formaldehyde and acrolein, both of which are highly cytotoxic to hepatocytes [120, 121]. Flavoring agents, though generally recognized as safe for ingestion, can become highly toxic upon inhalation following thermal degradation; for instance, cinnamaldehyde, a widely used flavoring compound, exhibits dose-dependent cytotoxicity in hepatocyte cultures even in the absence of nicotine [119]. Additionally, metallic elements, including chromium, nickel, and lead, are released from the device’s heating coils during aerosolization, introducing additional hepatotoxic risk [122].

Thus, while e-cigarettes eliminate many combustion-related toxins present in conventional cigarettes, they still emit a complex array of hazardous chemicals, including nicotine, volatile organic compounds, nitrosamines, polycyclic aromatic hydrocarbons (PAHs), aldehydes, and heavy metals [123]. These toxicants are rapidly absorbed via the pulmonary circulation, distributed systemically, and delivered to the liver, where they exert toxic effects [124].

### Hepatic effects and mechanisms of injury

The liver, being the primary site for xenobiotic metabolism and lipid/glucose regulation, is highly susceptible to the systemic effects of vaping aerosols. Evidence suggests multiple interrelated pathways of liver injury:

*Nicotine-induced injury*: Nicotine is metabolized in the liver via CYP2A6, where it activates α7-nicotinic acetylcholine receptors (α7-nAChR), leading to oxidative stress, mitochondrial dysfunction, and pro-fibrotic signaling [36, 125, 126].

*Oxidative stress*: Aerosolized chemicals and metals generate reactive oxygen species (ROS), depleting hepatic antioxidants (e.g., glutathione). This oxidative burden results in lipid peroxidation, DNA damage, and hepatocyte apoptosis [127, 128].

*Dysregulated lipid metabolism*: E-cigarette exposure alters key genes controlling lipid homeostasis (e.g., SREBP-1, ACC, FAS for synthesis; PPAR-α for oxidation), causing triglyceride accumulation and steatosis [129].

*Inflammatory cascade*: Vapors upregulate pro-inflammatory cytokines (TNF-α, IL-6, IL-1β) within hepatic tissue, driving steatohepatitis and early fibrosis [120, 130].

*Mitochondrial dysfunction and DNA damage*: Dysregulation of the NAD⁺/PARP1/SIRT1 axis and mitochondrial injury promotes fibrogenesis and hepatocyte dysfunction [131, 132].

### Evidence from experimental and clinical studies

#### Preclinical data

Animal studies have demonstrated that chronic exposure to e-cigarette vapor induces hepatic triglyceride accumulation and lipid droplet formation, hallmark features of MASLD [133]. In one experimental study, female mice, but not their male counterparts, showed significant elevations in hepatic triglyceride and phosphatidylcholine levels after exposure to e-vapor composed exclusively of glycerol, without nicotine or flavoring agents [121]. Furthermore, studies using diet-induced models of MASLD have shown that e-cigarette exposure exacerbates preexisting liver injury, worsening steatohepatitis through amplified oxidative stress, mitochondrial dysfunction, and inflammatory responses [134].

Animal experiments have further revealed transgenerational effects of vaping constituents. Maternal exposure to e-cigarette vapor during pregnancy disrupts nutrient metabolism and impairs liver health in both dams and their offspring. Notably, exposure to nicotine-free e-vapor induces significant metabolic alterations and liver injury in dams and progeny, whereas nicotine-containing e-vapor promotes hepatic steatosis in adult offspring [135].

#### Human evidence

Although human evidence remains limited due to the relatively recent rise in e-cigarette use, epidemiologic studies are beginning to corroborate preclinical findings. Analyses of the National Health and Nutrition Examination Survey (NHANES) have revealed that current e-cigarette users have a significantly higher odds of MASLD compared to nonusers [136]. E-cigarette use has been linked to metabolic dysfunction, including prediabetes and impaired fasting glucose regulation, suggesting broader systemic metabolic disruption [137].

### Dual use

The dual use of e-cigarettes and traditional combustible cigarettes, a pattern increasingly observed worldwide, poses compounded risks for metabolic and liver health [138, 139]. Dual users are exposed to a higher cumulative burden of hepatotoxicants, which may accelerate the progression of liver disease. Clinical evidence indicates that dual users have significantly greater odds of developing metabolic syndrome, elevated triglyceride levels, and reduced HDL-cholesterol compared to never smokers [136].

Cross-sectional studies further support this concern, showing a significant association between dual use and MASLD. In one study, adjusted odds ratios for MASLD demonstrated a clear gradient: lowest in never smokers, higher in exclusive cigarette smokers, and highest among dual users, suggesting an additive or even synergistic effect of combined exposure [140]. This pattern is particularly alarming in individuals with existing metabolic risk factors or liver disease, where dual use may exacerbate hepatic steatosis, inflammation, and fibrosis.

### Conclusion

The current evidence clearly indicates that e-cigarette use is not harmless to the liver. It is strongly associated with the development and progression of hepatic steatosis, increased oxidative stress, mitochondrial dysfunction, activation of pro-inflammatory and pro-fibrotic pathways, and systemic metabolic dysregulation. These mechanisms collectively contribute to the pathogenesis and worsening of MASLD. Although e-cigarettes eliminate some combustion-related toxins found in conventional cigarettes, they introduce a unique array of hepatotoxic compounds that may exert additive or synergistic effects when combined with traditional smoking.

## Smoking cessation and clinical implications

The overwhelming evidence presented herein underscores the critical importance of smoking cessation as a fundamental and highly impactful intervention in the prevention and management of liver diseases. Given the pervasive and multifaceted detrimental effects of tobacco on liver health, promoting and facilitating smoking cessation should be a cornerstone of clinical practice for all healthcare professionals, particularly hepatologists and gastroenterologists.

### Benefits of smoking cessation on liver disease outcomes

Smoking cessation offers substantial benefits across the spectrum of liver diseases. For patients with MASLD, quitting smoking can mitigate the progression of fibrosis and reduce the risk of cardiovascular events, which are the leading cause of mortality in this population [62, 141, 142]. While smoking cessation may initially be associated with weight gain, potentially influencing MASLD development, the overall benefits far outweigh this risk, provided patients are monitored by a multidisciplinary team including dieticians [143, 144].

In the context of viral hepatitis, smoking cessation can improve treatment response rates to antiviral therapies and slow down the progression of fibrosis and inflammation [88, 145]. Furthermore, for patients with ALD, cessation can reduce the synergistic harm caused by combined alcohol and tobacco exposure and may decrease the likelihood of alcohol relapse post-transplant [115]. For individuals at risk of or diagnosed with HCC, quitting smoking significantly reduces the risk of developing this aggressive cancer and can improve survival outcomes [105, 146]. The reduction in HCC risk for ex-smokers compared to current smokers is a powerful incentive for cessation [101].

For patients undergoing or being considered for liver transplantation, smoking cessation is of paramount importance. Quitting smoking significantly lowers the risk of post-transplant complications, including vascular events and de novo malignancies [108, 147–149]. Evidence also indicates that cessation efforts improve both patient and graft survival, highlighting the need for comprehensive smoking cessation programs and supportive measures within transplant centers [113].

### Challenges in promoting smoking cessation in liver disease patients

Despite the clear benefits, promoting smoking cessation in patients with liver disease presents several challenges. A significant barrier is the often underestimated awareness among healthcare providers regarding the direct link between smoking and liver disease [150]. A survey revealed that less than 25% of hepatologists refer smokers to specialized cessation units, indicating a gap in integrating smoking cessation into routine liver care [151]. This lack of proactive engagement can stem from various factors, including time constraints, insufficient training in smoking cessation counseling, and a focus on other prominent risk factors for liver disease. This highlights a critical gap in clinical practice, emphasizing the need for hepatologists and gastroenterologists to be more proactive in identifying smoking as a risk factor for liver disease progression and in implementing strategies to promote smoking cessation among their patients.

Patients themselves may also face considerable hurdles. Nicotine dependence is a powerful addiction, and many patients with liver disease may have co-occurring substance use disorders, particularly alcohol dependence, which complicates cessation efforts [76]. The psychological and social aspects of smoking, coupled with potential withdrawal symptoms, can make quitting extremely difficult without adequate support. Additionally, concerns about weight gain post-cessation, particularly relevant for MASLD patients, can deter individuals from attempting to quit [152].

### Role of hepatologists and multidisciplinary teams

**Fig. 3:**
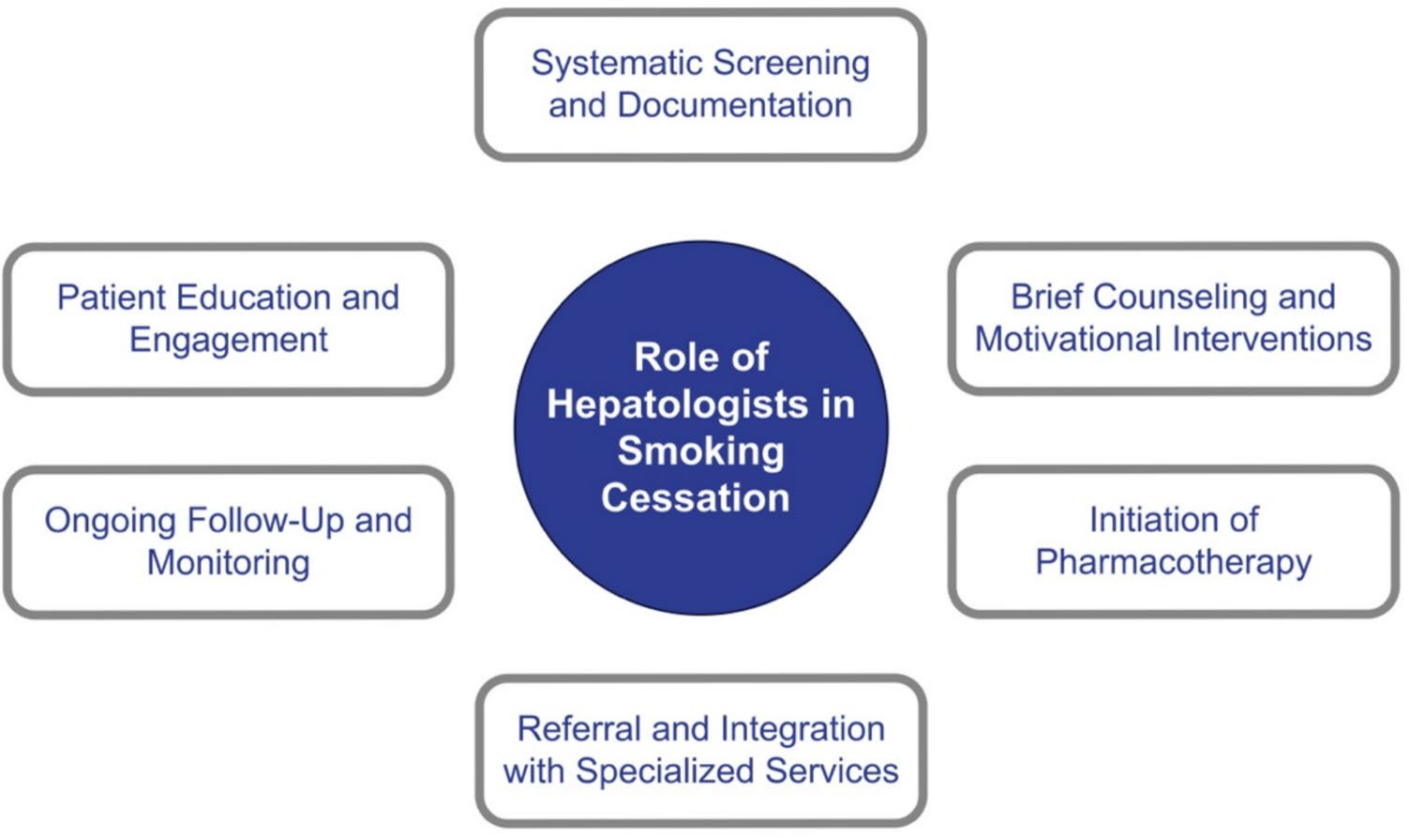
Role of hepatologist in smoking cessation. A schematic overview illustrating the key responsibilities of hepatologists in integrating smoking cessation into liver disease management

Hepatologists, as primary specialists managing chronic liver disease, are uniquely positioned to identify and address tobacco use as a modifiable risk factor. Their role extends beyond diagnosis and treatment to actively supporting smoking cessation as an integral component of liver care. To achieve this, hepatologists should implement structured, evidence-based strategies within routine clinical practice (Fig. 3).

#### Systematic screening and documentation

Every patient with liver disease should undergo standardized assessment of smoking status at initial and follow-up visits. This includes documenting current, former, or never-smoking status, type of tobacco product (traditional cigarettes, e-cigarettes), quantifying pack-years, and evaluating secondhand smoke exposure. E-cigarette use should be routinely assessed and recognized as a modifiable risk factor. Incorporating these details into electronic health records ensures continuity of care and facilitates progress tracking [3].

#### Brief counseling and motivational interventions

Hepatologists should provide concise, structured counseling that emphasizes the impact of smoking on liver health, including accelerated fibrosis progression, reduced treatment response, and increased hepatocellular carcinoma risk. Patients should also be educated on the hepatotoxic potential of e-cigarettes. Motivational interviewing techniques can strengthen patient commitment by linking cessation to improved liver outcomes and overall health benefits [153].

#### Initiation of pharmacotherapy

When appropriate, hepatologists should initiate first-line pharmacologic treatments such as nicotine replacement therapy (NRT), varenicline, or bupropion, all of which have proven efficacy in smoking cessation. These medications should be integrated into the overall liver disease management plan, with careful consideration of hepatic function and potential drug interactions [154, 155].

#### Referral and integration with specialized services

Clear referral pathways to specialized smoking cessation programs are essential. These programs can provide behavioral counseling, pharmacologic optimization, and relapse prevention strategies. A multidisciplinary team, including dietitians, addiction specialists, psychologists, and social workers, can address coexisting challenges such as anxiety, depression, and weight gain during the cessation process [150].

#### Ongoing follow-up and monitoring

Support should continue beyond initial counseling. Hepatologists should schedule structured follow-up visits or telehealth sessions to monitor abstinence, adjust pharmacotherapy, and reinforce behavioral interventions. Integrating smoking cessation progress into liver disease monitoring (e.g., fibrosis staging, treatment outcomes) can help sustain long-term success.

#### Patient education and engagement

Patient empowerment through education is critical. Clinicians should clearly explain how smoking accelerates liver disease progression and how cessation reduces the risk of cirrhosis, hepatocellular carcinoma, and transplantation complications. Visual aids, decision-support tools, and digital resources can further enhance understanding and adherence.

By incorporating these strategies into standard hepatology practice, healthcare providers can significantly increase smoking cessation rates among patients with liver disease, leading to improved clinical outcomes and reduced healthcare burden. The future research should focus on evaluating the effectiveness of hepatology-led cessation interventions and their long-term impact on liver health.

## Future directions

Future research should aim to elucidate the molecular and cellular pathways by which tobacco smoke and vaping constituents drive liver injury, immune dysregulation, and carcinogenesis. Priority areas include studying the long-term impact of smoking cessation on fibrosis regression, HCC risk reduction, and overall survival, as well as identifying genetic and epigenetic factors that confer heightened susceptibility to smoking-related liver damage.

Given the rising global prevalence of e-cigarette and heated tobacco product use, particularly among younger populations, there is an urgent need for longitudinal studies assessing their hepatotoxic potential, including device- and flavor-specific effects. Investigating transgenerational impacts, such as those observed in animal studies, should also be prioritized. Additionally, clinical trials evaluating tailored behavioral and pharmacologic cessation interventions for patients with liver disease are essential to inform evidence-based guidelines.

## Conclusion

The cumulative evidence strongly establishes cigarette smoking as a major and independent risk factor for the onset, progression, and adverse outcomes of multiple liver diseases. Smoking accelerates hepatic steatosis, promotes fibrosis, increases the risk of HCC, and compromises post-transplant outcomes. These detrimental effects are mediated by complex mechanisms, including direct hepatotoxicity, oxidative stress, immune dysregulation, oncogenic signaling, and systemic metabolic disturbances.

Importantly, e-cigarettes are not a safe alternative. Although they eliminate some combustion-derived toxins, they still deliver harmful substances capable of inducing oxidative stress, mitochondrial dysfunction, inflammation, and hepatocellular injury. Current evidence suggests that both traditional and electronic smoking contribute to metabolic dysregulation and fibrosis progression, with dual users potentially facing compounded risks.

Despite compelling data, awareness of the link between smoking and liver disease remains suboptimal among healthcare providers and the general public. Smoking and vaping cessation should therefore be considered essential components of liver disease management. This requires systematic screening, structured counseling, integration of pharmacologic therapies, and referral to specialized cessation services supported by a multidisciplinary approach.

## Data Availability

No datasets were generated or analysed during the current study.

## Abbreviations

aHR: Adjusted hazard ratio
ALT: Alanine aminotransferase
ALD: Alcohol-related liver disease
CV: Cardiovascular disease
CYP1A2: Cytochrome P450 1A2
HBV: Hepatitis B virus
HCV: Hepatitis C virus
HCC: Hepatocellular carcinoma
HR: Hazard ratio
HSCs: Hepatic stellate cells
IL: Interleukin
LT: Liver transplantation
MASLD: Metabolic dysfunction-associated steatotic liver disease
MAPKs: Mitogen-activated protein kinases
MetS: Metabolic syndrome
NDMA: N-nitrosodimethylamine
NF-κB: Nuclear factor-kappa B
NHANES: National Health and Nutrition Examination Survey
NRT: Nicotine replacement therapy
OR: Odds ratio
PAHs: Polycyclic aromatic hydrocarbons
PBC: Primary biliary cholangitis
PG: Propylene glycol
ROS: Reactive oxygen species
RR: Relative risk
TERT: Telomerase reverse transcriptase
TGF-β1: Transforming growth factor beta 1
TNF-α: Tumor necrosis factor alpha
VG: Vegetable glycerin
α7-nAChR: α7-Nicotinic acetylcholine receptor
β2-AR: β2-Adrenergic receptor
